# Development of Conventional Multiplex PCR: A Rapid Technique for Simultaneous Detection of Soil-Transmitted Helminths

**DOI:** 10.3390/pathogens8030152

**Published:** 2019-09-16

**Authors:** Vivornpun Sanprasert, Ruthairat Kerdkaew, Siriporn Srirungruang, Sarit Charuchaibovorn, Kobpat Phadungsaksawasdi, Surang Nuchprayoon

**Affiliations:** 1 Lymphatic Filariasis and Tropical Medicine Research Unit, Chulalongkorn Medical Research Center, Faculty of Medicine, Chulalongkorn University, Bangkok 10330, Thailand; vivornpun@chula.md (V.S.); joruthairat@gmail.com (R.K.); lingnooy55@windowslive.com (S.S.); sarit.src@gmail.com (S.C.); 2 Department of Parasitology, Faculty of Medicine, Chulalongkorn University, Bangkok 10330, Thailand; kobpat22@hotmail.com; 3 Department of Parasitology, King Chulalongkorn Memorial Hospital, Bangkok 10330, Thailand

**Keywords:** multiplex PCR, PCR, soil-transmitted helminths (STHs), diagnosis

## Abstract

Soil-transmitted helminths (STHs) are the most common intestinal parasites infecting humans worldwide. STH infections are a major cause of morbidity and disability. Accurate diagnostic tools are pivotal for assessing the exact prevalence of parasitic infections. Microscopic examination and culture techniques have been used to observe the presence of eggs or larvae of parasites in stool samples, but they are time-consuming and have low sensitivity. Therefore, accurate, simple, and inexpensive diagnostic techniques are still required for simultaneous detection of STH infections. Although molecular-based techniques, such as real-time PCR and multiplex real-time PCR, have been developed, they are not suitable for routine diagnosis due to the requirement for expensive reagents and instruments. In this study, we established a conventional multiplex PCR for simultaneous rapid detection of *Ascaris lumbricoides*, *Necator americanus*, and *Strongyloides stercoralis* in stool samples. Our results show that the multiplex PCR could detect the DNA of STHs at a very low target gene concentrations (lower than 1 pg) with no cross-amplification. Multiplex PCR had five times higher sensitivity than the formalin–ethyl acetate concentration technique (FECT) in the detection of multiple infections, and two times higher for detection of *S. stercoralis*. However, multiplex PCR was comparable to FECT in the detection of *A. lumbricoides* and *N. americanus*. In conclusion, this method could be used as an alternative method for the detection of STHs, especially for *S. stercoralis*.

## 1. Introduction

Soil-transmitted helminths (STHs) or geohelminths pose a major public health threat to at least 2 billion people each year around the world. STHs are a group of parasitic nematodes including *Ascaris lumbricoides* (807–1221 million people), hookworms (*Necator americanus* and *Ancylostoma duodenale*; 564–740 million people), *Strongyloides stercoralis* (30–100 million people), and *Trichuris trichiura* (604–795 million people) [1,2]. STH infections are associated with malnutrition, cognitive impairment, and health problems (e.g., intestinal obstruction and anemia) in children, as suggested by the World Health Organization (WHO). These parasites are typically found in tropical and sub-tropical countries where sanitation and hygiene are lacking, especially in low- and middle-income countries [1]. These parasites are easily transmitted and co-infected via the fecal–oral route (food or water containing infective eggs or infective larvae) or skin surface contact (hookworms and *S. stercoralis*) [1,3,4].

Accurate diagnostic tools play a pivotal role in epidemiological study, monitoring of treatment efficacies in mass drug administration (MDA) programs, and monitoring the possibility of drug-resistance development. The traditional diagnostic techniques for STH infections include microscopic examinations of stool samples and a culture technique. The Kato–Katz fecal thick smear is the most common method recommended by WHO. However, the Kato–Katz technique has limitations, especially a low sensitivity in light infection individuals and low sensitivity when using a single Kato–Katz thick smear [5,6,7]. Moreover, this technique has poor sensitivity for *S. stercoralis* infections [8,9,10].

DNA-based techniques have been developed for the detection and differentiation of intestinal parasites in fecal samples [11]. These include polymerase chain reaction (PCR), nested PCR, PCR-restriction fragment length polymorphism (PCR-RFLP), and quantitative PCR. The loop-mediated isothermal amplification assay (LAMP) is a one-step assay used to amplify target DNA under a constant temperature without the need for a thermal cycler. The LAMP result can be read with the naked eye. LAMP has been widely adapted for the rapid detection of helminths, and has shown high sensitivity and specificity [12]. However, several reactions using specific primers for each parasite need to be used to diagnose STH infections. Multiplex LAMP (mLAMP) technology has been explored more recently. However, the major drawbacks of LAMP and mLAMP are the difficulties of the primer design and the test evaluation. Multiplex real-time PCR and digital multiplex PCR (dmPCR) have also been proposed for highly sensitive and specific simultaneous detection of parasites [6,9,10,11,12,13,14,15]. However, they are limited by the requirement of expensive reagents and instruments for result interpretation. Therefore, these advanced techniques are not suitable for the developing countries that are the endemic areas of STHs. Therefore, conventional multiplex PCR is a simple and cost-effective method that can be used to detect mixed infections in a single reaction. Several multiplex PCR assays have been developed for the detection of parasites [13,14,15]. In this study, we developed a conventional multiplex PCR for simultaneous infections of *A. lumbricoides*, *N. americanus*, and *S. stercoralis,* providing an efficient and convenient technique for epidemiological and clinical studies.

## 2. Results

### 2.1. Species-Specific Primers of Each Parasite to Specifically Amplify Target Amplicons

**Figure 1:**
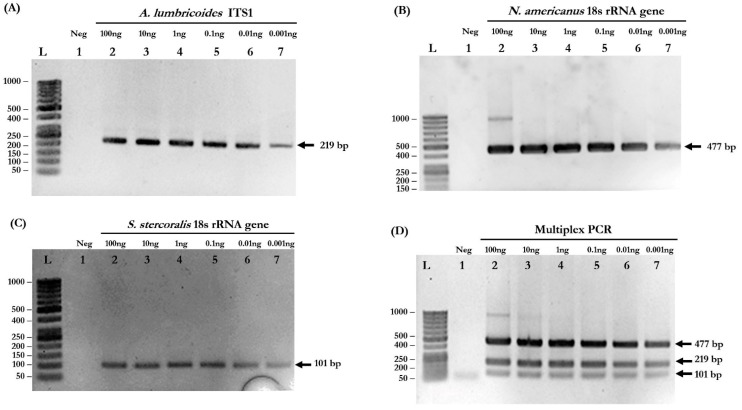
Determination of the detection limits of simplex and multiplex PCR. (**A**) Detection of *A. lumbricoides* ITS1 gene. Lane L: DNA ladder; Lane 1: negative control; Lanes 2–7: 10-fold serial dilutions of gDNA of *A. lumbricoides* at 100, 10, 1, 0.1, 0.01, and 0.001 ng. (**B**) Detection of *N. americanus* 18s rRNA gene. Lane L: DNA ladder; Lane 1: negative control; Lanes 2–7: 10-fold serial dilutions of gDNA of *N. americanus* at 100, 10, 1, 0.1, 0.01, and 0.001 ng. (**C**) Detection of *S. stercoralis* 18s rRNA gene. Lane L: DNA ladder; Lane 1: negative control; Lanes 2–7: 10-fold serial dilutions of gDNA of *S. stercoralis* at 100, 10, 1, 0.1, 0.01, and 0.001 ng. (**D**) The sensitivity of multiplex PCR. Lane L: DNA ladder; Lane 1: negative control; Lanes 2–7: 10-fold serial dilutions of mixed gDNA at 100, 10, 1, 0.1, 0.01, and 0.001 ng of each parasite.

Based on the simplex PCR results, the optimized conditions for multiplex PCR were determined: primer concentration = 0.16 µM, MgCl_2_ concentration = 3 mM, and annealing temperature = 58 °C. The primers used in this study are listed in Table 1. Agarose gel electrophoresis confirmed that the PCR products were in the correct sizes. The target amplicons of each species-specific primer, including 220 base pairs (bp) for *A. lumbricoides*, 483 bp for *N. americanus*, and 100 bp for *S. stercoralis,* were obtained from simplex PCR (Figure 1A–C, respectively). In the multiplex PCR reaction, all three different-sized amplicons were simultaneously amplified (Figure 1D).

### 2.2. Multiplex PCR Is Sensitive for Simultaneous Detection of Mixed Infections

To evaluate the detection limits of simplex and multiplex PCR, genomic DNA isolated from each pure parasite was serially diluted 10-fold to the range of 100 to 0.001 ng. Simplex PCR reactions were able to detect 0.001 ng of *A. lumbricoides*, *N. americanus*, and *S. stercoralis* (Figure 1A–C, respectively). The multiplex PCR reaction was also able to detect all STH species at a very low concentration of gDNA (0.001 ng) (Figure 1D). The PCR products of each STH were cloned into pGEM^®^-T Easy Vector and used as “plasmid controls” in the next experiments.

### 2.3. No Cross-Reactivity of Each Species-Specific Primer and Other Parasites

**Figure 2:**
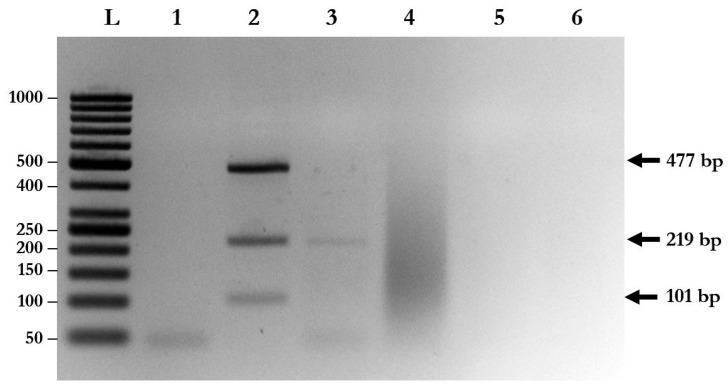
Determination of the specificity of multiplex PCR. Lane L: DNA ladder; Lane 1: negative control; Lane 2: multiplex PCR using triple plasmid controls; Lane 3: multiplex PCR using gDNA isolated from *A. lumbricoides and Giardia lamblia*; Lane 4: multiplex PCR using gDNA isolated from *G. lamblia*; Lane 5: multiplex PCR using gDNA isolated from *G. lamblia* and *Blastocystis hominis*; Lane 6: parasite-free human feces.

Species-specific primers of *A. lumbricoides, N. americanus*, and *S. stercoralis* were used to evaluate the specificity of multiplex PCR with triple plasmid controls. The results show that each pair of species-specific primers could not cross-amplify with the plasmids and with other parasite species (Figure 2). Moreover, cross-amplification with other intestinal parasites, such as *Enterobius vermicularis* (data not shown), *Giardia lamblia*, and *Blastocystis hominis*, was not found with multiplex PCR (Figure 2).

### 2.4. Multiplex PCR Correctly Identifies Parasites in Stool Samples 

**Figure 3:**
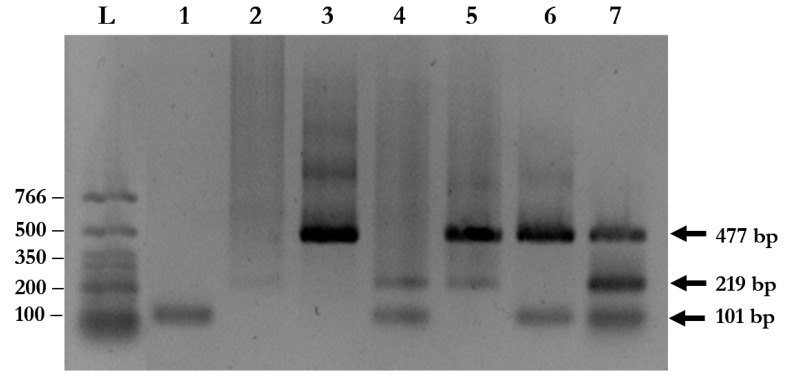
Multiplex PCR for simultaneous detection of soil-transmitted helminth (STH) infections using clinical samples. The amplified fragments from species-specific primers were visualized on 1% agarose gel using ethidium bromide staining. Lane L: DNA ladder; Lane 1: *S. stercoralis*; Lane 2: *A. lumbricoides*; Lane 3: *N. americanus*; Lane 4: *S. stercoralis* and *A. lumbricoides*; Lane 5: *N. americanus* and *A. lumbricoides*; Lane 6: *S. stercoralis* and *N. americanus*; and Lane 7: *S. stercoralis*, *A. lumbricoides*, and *N. americanus.*

**Figure 4:**
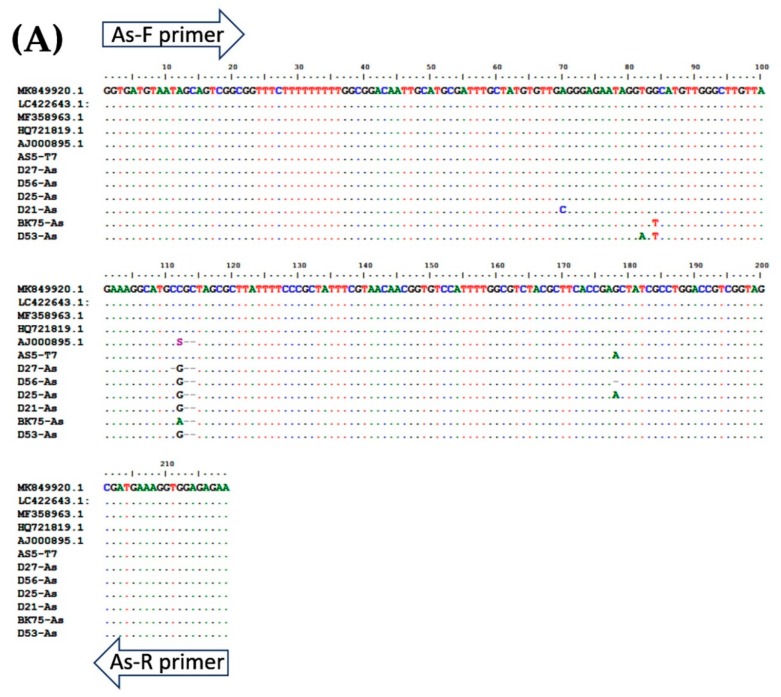

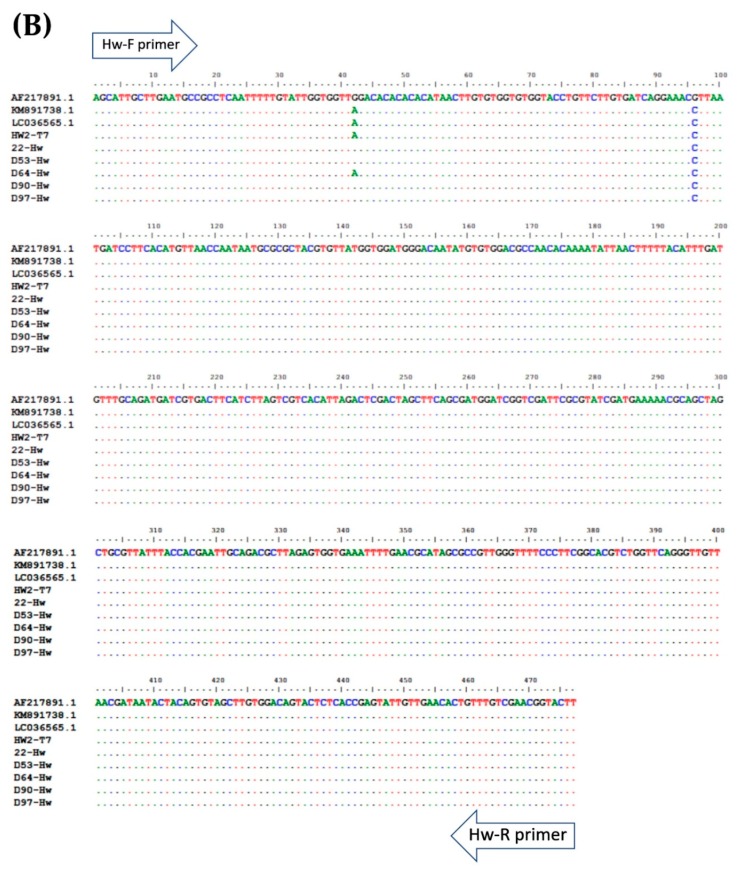

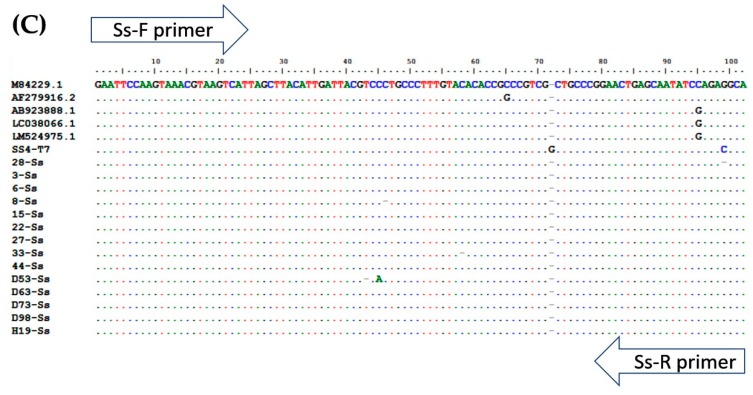
Multiple alignment of PCR amplicons with plasmid controls and published sequences in the GenBank Database: (**A**) *A. lumbricoides*; ITS1 (Accession No. AJ000895.1), (**B**) *N. americanus*; 18s rRNA (Accession No. AF217891.1), and (**C**) and *S. stercoralis*; 18S rRNA (Accession No. AF279916.2). Perfect matches to the reference sequence are shown as dots. Mismatches are shown as capital letters. Sequence gaps are shown as hyphens.

A total of 94 stool samples collected from patients presenting with gastrointestinal symptoms were recruited for the study. The multiplex PCR assay could identify single, double, and triple infections of *A. lumbricoides*, *N. americanus*, and *S. stercoralis* (Figure 3 and Table 2). Agarose gel electrophoresis and DNA sequencing of the purified PCR amplicons confirmed that the PCR products were the corrected sizes, with 98.04–100% identity to the published sequences in the GenBank Database (Figure 4, Table S1).

### 2.5. Comparison of Multiplex PCR and FECT for Detection of STHs in Stool Samples 

Out of 94 samples, 49 samples (52.13%) were positive for STHs and 45 samples (47.87%) were negative for FECT (Table 2). FECT detected 47 individuals (50.0%) with a single infection, and 2 individuals (2.13%) with a double infection, including co-infection between *A. lumbricoides* and *N. americanus*. There was no positive result for triple infection detected by FECT (Table 2).

Compared with FECT, 66 samples (70.21%) resulted positive for STHs and only 28 samples (29.79%) were negative from multiplex PCR (Table 2). The multiplex PCR assay detected 55 individuals (58.51%) with a single infection, 9 individuals (16.67%) with a double infection, and 2 individuals (2.13%) with a triple infection (Table 2). Overall, the detection rate of multiplex PCR was significantly higher than that of FECT (66 of 94; 70.21% vs. 49 of 94; 52.13%; *p* = 0.0109). The multiplex PCR had a 5.5-fold greater success rate in the detection of multiple infections than FECT (Table 2).

The detection rate of *A. lumbricoides* by multiplex PCR was non-significantly higher than FECT (34 of 94; 36.17% vs. 28 of 94; 29.78%; *p* = 0.3514) (Table 3). The detection rate of *N. americanus* by multiplex PCR was comparable with that of FECT (7 of 94; 7.45% vs. 7.45%). The detection rate of *S. stercoralis* by multiplex PCR was significantly—2.4 times—higher than FECT, (38 of 94; 40.43% vs. 16 of 94; 17.02%; *p =* 0.0026) (Table 3).

For detection of *A. lumbricoides*, multiplex PCR showed substantial agreement (*κ* = 0.617) with FECT. However, the agreement between FECT and multiplex PCR was fair for detection of *N. americanus* (*κ* = 0.383) and *S. stercoralis* (*κ* = 0.318) (Table 3).

## 3. Discussion

STH infections are a major public health problem for 1.5 billion of the world’s population in tropical and sub-tropical areas, with the greatest number in Southeast Asia, Sub-Saharan Africa, and South America [3]. STH infections usually cause morbidity and disability to infected individuals, especially children. Malnutrition, malabsorption syndrome, intestinal obstruction, poor weight gain, and iron-deficiency anemia are common morbidities of STH infections [3,16]. Disseminated infection with *S. stercoralis* may be fatal and life-threatening [17]. Co-infection of STHs is usually found [18,19]. The efficacy, dose, and timing of anthelmintic treatment depend on the helminth species, although the mild symptoms of STHs are similar. Therefore, the simultaneous diagnosis of STH infections is required.

Kato–Katz thick smear, direct smear, FECT, and FLOTAC are commonly used for detection of the eggs of *A. lumbricoides*, hookworms, and *T. trichiura*. Although these techniques are simple and inexpensive, they often suffer from low sensitivity, especially in individuals with light infections. They also require a microscopy technician to prevent a false negative result. Agar plate culture is the strongest technique for *S. stercoralis* and hookworm diagnosis [18,19]. However, it is time-consuming (five to seven days) and often produces misdiagnoses [18]. Multiplex real-time PCR for simultaneous detection of STHs and other parasites has been established [20,21,22,23,24]. This technique provides high sensitivity and specificity.

In this study, we developed a conventional multiplex PCR. Our multiplex PCR is capable of simultaneous detection of *A. lumbricoides*, *N. americanus*, and *S. stercoralis*. Using a pair of species-specific primers, amplified fragments with different sizes were obtained. Our results showed a low detection limit, down to 0.001 ng of DNA template. Simplex PCR, with the multiplex PCR condition, was able to amplify gDNA isolated from worms at very low concentrations (0.001 ng) for all parasites (Figure 1A–C).

For the multiplex PCR reaction, our results showed a high efficiency for simultaneous detection of STH infections at very low concentrations (0.001 ng; Figure 1D). Neither cross-reactivities between each species-specific primers nor the genomic DNA of other parasites, including *E. vermicularis*, *G. lamblia*, and *B. hominis*, were found. However, cross-reactivity in other human intestinal parasites should be further investigated. In this study, 25 stool samples of *S. stercoralis* resulted positive for multiplex PCR but negative in microscopic examinations, suggesting that the multiplex PCR can detect *S. stercoralis* at a rate up to two times higher than by FECT. This indicates that this tool has a high sensitivity for the detection of *S. stercoralis.*

Unfortunately, we found that 12 samples that were identified as positive by FECT were negative by multiplex PCR. This might be due to several factors, such as DNA degradation during storage and transportation, non-homogeneity of eggs and larvae in stool samples, and a DNA isolation method that was not efficient enough to destroy the thick egg shells or larvae. Moreover, the presence of PCR inhibitors in stool samples can cause false negatives in multiplex PCR. PCR reactions with diluted DNA template, human internal controls, and plasmid spike tests could be performed in future studies to confirm the absence of PCR inhibitors in the stool samples.

In this study, we used only FECT to detect STH infections. Therefore, we could not determine the exact sensitivity and specificity of multiplex PCR. Although Kato–Katz is the most common technique for detection of STH infections, a previous study showed that formalin–ether concentration technique had a higher sensitivity than the Kato–Katz technique based on a single stool sample for STH infections *T. trichiura*, hookworms, and *A. lumbricoides* [7]. In our study, we used ethyl acetate instead of ether, because it is more safe and shows comparable results to the formalin–ether concentration technique [25]. However, quantitative FECT had lower sensitivity in the detection of S. *stercoralis* compared to agar plate culture, especially when the parasite intensity was lower than 50 larvae per gram of stool [26]. In this study, we showed that multiplex PCR was more effective than FECT for the detection of *S. stercoralis* even in patients who had low parasite intensity or resulted negative for FECT. We also designed specific primers for *A. duodenale* (data not shown). Due to the lack of this parasite in Thailand, we could not perform multiplex PCR to differentiate between *A. duodenale* and *N. americanus*.

In conclusion, our results indicate that multiplex PCR is a simple, rapid, and cost-effective method for the simultaneous detection of STHs infections, including *A. lumbricoides, N. americanus*, and *S. stercoralis*. The cost of each multiplex PCR reaction followed by agarose gel electrophoresis was less than USD $2. This multiplex PCR is therefore a potential alternative method that could be convenient and reliable for epidemiological and clinical studies.

## 4. Materials and Methods 

### 4.1. Sample Collection and Examination

Ninety-four stool samples were collected from patients presenting with gastrointestinal symptoms from the Laboratory of Parasitology, King Chulalongkorn Memorial Hospital, Bangkok, Thailand. The presence of eggs or larvae of parasites was confirmed in each sample using the formalin–ethyl acetate concentration technique (FECT) [27]. The remaining stool samples of each individual were preserved at −20 °C until DNA extraction. An adult worm of *A. lumbricoides* was collected from a stool sample and cut into small pieces. An adult worm of *N. americanus* was obtained from a patient during colonoscopy. Two hundred filariform larvae (L3s) of *S. stercoralis* were collected from the agar plate culture. Worms were washed with sterile normal saline and chopped up on ice with a sterile scalpel blade. The chopped-up worm tissue fragments were homogenized in a pestle homogenizer and ice-chilled with sterile normal saline. The parasite homogenates were stored at −70 °C until used. This study was approved by the Human Ethics Committees of the Faculty of Medicine, Chulalongkorn University (IRB No. 483/58).

### 4.2. DNA Preparation

The genomic DNA was isolated from parasite homogenates and stool samples based on a phenol–chloroform method with some modifications. Briefly, the parasite homogenates or 250 mg of stool samples were resuspended with lysis buffer (20 mM Tris-HCl pH 7.6, 2.5 mM MgCl_2_, 50 mM KCl, 150 µg/mL proteinase K, 0.5% Tween-20) and were then incubated at 65 °C for 3 hours, followed by 90 °C for 10 min to inactivate proteinase K. Two hundred microliters of phenol, chloroform, and isoamyl alcohol (ratio 25:24:1) were added and mixed by inverting the tube. The mixture was then centrifuged at 13,000 rpm at 4 °C for 10 min. Upper aqueous phases were transferred to a new 2 mL tube containing two volumes of chloroform, and were then centrifuged at 13,000 rpm at 4 °C for 10 min. Genomic DNA was precipitated from the upper phases using 2.5 volumes of ice-cold absolute ethanol and 0.1 volumes of 3 M sodium acetate at −20 °C overnight. A pellet of genomic DNA was washed with 70% ethanol and finally resuspended with nuclease-free water. The concentration and purity of the genomic DNA were determined using a Nanodrop 1000 spectrophotometer (Thermo Scientific, Waltham, MA, USA).

### 4.3. Primer Design for Multiplex PCR

To perform multiplex PCR, each pair of species-specific primers was selected from target genes, including *A. lumbricoides*; ITS1 (accession No. AJ000895.1), *N. americanus*; 18s rRNA (accession No. AF217891.1), and *S. stercoralis*; 18S rRNA (accession No. AF279916.2). Specific primers for *A. lumbricoides* were designed using the Primer 3 program. Specific primers for *N. americanus* and for *S. stercoralis* were modified from previous studies [28,29]. The target amplicons were generated as different sizes. Pairs of multiple primers were analyzed and compared using a multiple primer analyzer (Thermo Scientific, Waltham, MA, USA). The primer sequences and product sizes are described in Table 1.

### 4.4. Multiplex PCR

First, simplex PCR was performed to optimize the reaction of each pair of species-specific primers by adjusting a range of concentrations of Mg^2+^, annealing temperature, and concentration of each primer. All of the species-specific primers were then used to conduct multiplex PCR based on the optimization results of the simplex PCR. The MgCl_2_ concentration and annealing temperature were adjusted to obtain the optimal conditions for multiplex PCR. The optimized multiplex PCR mixture was then conducted in a 20 µL reaction. Multiplex PCR was performed under the following conditions: 94 °C for 5 min, 30 cycles at 94 °C for 1 min, at 58 °C for 1 min, and at 72 °C for 1 min, followed by a final extension at 72 °C for 5 min. The results were determined by 1% agarose gel electrophoresis in 1× TAE buffer for 30 min. The gel was stained with ethidium bromide to visualize the DNA fragments under a UV transilluminator.

### 4.5. Determination of Specificity and Limit of Detection 

To determine the specificity of each primer pair, gDNA isolated from adult worms of *A. lumbricoides*, *N. americanus*, and L3s of *S. stercoralis* were amplified by multiplex PCR (Figure 1) singly and in combinations of two and three. Subsequently, the PCR products of each STH were cloned into pGEM^®^-T Easy Vector (Promega, Madison, WI, USA). After the transformation and screening of the clones, selected positive clones were sent for DNA sequencing. The plasmids were sequenced in both directions using T7 primers (U2Bio Sequencing Service, Bangkok, Thailand). The sequences of the PCR products were processed using BioEdit Sequence Alignment Editor version 7.2.5 [30] and compared with sequences of closely-related species in GenBank by ClustalW Multiple Alignment (Figure 4). Theses recombinant plasmids were used as the “plasmid control” in multiplex PCR.

To determine the limit of detection, 0.001–100 ng of gDNA isolated from each parasite was used as a template for determination of the detection rate of each species-specific primer, for both simplex and multiplex PCR (Figure 1).

To evaluate the sensitivity and specificity, multiplex PCR was performed with gDNA isolated from stool samples collected from patients infected with *A. lumbricoides*, hookworm, *S. stercoralis*, and other intestinal parasites commonly found in Thailand, including *Enterobius vermicularis*, *Blastocystis hominis*, and *Giardia lamblia*. Negative control material (nuclease-free water) and plasmid controls were included with each run.

### 4.6. Statistical Analysis

Statistical analysis was performed using STATA statistical package version 14 (STAT Inc., Chicago, IL, USA). The degrees of agreement between multiplex PCR and FECT were determined by Cohen’s Kappa statistic. Proportional statistic test was used to compare detection rate of multiplex PCR and FECT. *P* values < 0.05 were considered statistically significant.

## Figures and Tables

**Table 1:** Oligonucleotide sequences for simultaneous detection of *Ascaris lumbricoides*, *Necator americanus*, and *Strongyloides stercoralis* by multiplex PCR. F: Forward, R: Reverse.

Parasite	Target Region (Accession No.)	Primer (5′→ 3′)	Length (bp)	Product Size (bp)
*A. lumbricoides*	ITS1	F: GGT GAT GTA ATA GCA GTC GG	20	219
	(AJ000895.1)	R: TTC TCT CCA CCT TTC ATC G	19	
*N. americanus*	18S rRNA	F: AGC ATT GCT TGA ATG CC	17	477
	(AF217891.1)	R: AAG TAC CGT TCG ACA AAC AG	20	
*S. stercoralis*	18S rDNA	F: GAATTCCAAGTAAACGTAAGTCAT	24	101
	(AF279916.2)	R: TGCCTCTGGATATTGCTCAGTTC	23	

**Table 2:** Comparison of multiplex-PCR and formalin–ethyl acetate concentration technique (FECT) for the detection of STH infections.

	Multiplex- PCR	FECT
**Positive**	**66**	**49**
Single infection		
*A. lumbricoides*	25	26
*N. americanus*	0	5
*S. stercoralis*	30	16
Co-infection		
*A. lumbricoides* and *N. americanus*	3	2
*A. lumbricoides* and *S. stercoralis*	4	0
*N. americanus* and *S. stercoralis*	2	0
*A. lumbricoides*, *N. Americanus*, and *S. stercoralis*	2	0
**Negative**	**28**	**45**

**Table 3:** Statistical analysis of comparison of multiplex PCR and FECT in the detection of *A. lumbricoides*, *N. americanus*, and *S. stercoralis*. “Pos” denotes positive, “Neg” denotes negative.

	Multiplex PCR	FECT		Total	Kappa
		Positive	Negative		
***A. lumbricoides***	Positive	23	11	34	0.617
	Negative	5	55	60	
	Total	28	66	94	
***N. americanus***	Positive	3	4	7	0.383
	Negative	4	83	87	
	Total	7	87	94	
***S. stercoralis***	Positive	13	25	38	0.318
	Negative	3	53	56	
	Total	16	78	94	

## Supplementary Materials

The following are available online at https://www.mdpi.com/2076-0817/8/3/152/s1, Table S1: Blast results for amplicons of the clinical samples and plasmid controls.
