# Supplementary material for: Development of Conventional Multiplex PCR: A Rapid Technique for Simultaneous Detection of Soil-Transmitted Helminths
Source: Pathogens. 2019 Sep 16;8(3):152. doi: 10.3390/pathogens8030152 (PMC6789620; doi:10.3390/pathogens8030152)
Supplement: Supplementary file 1 [file pathogens-08-00152-s001.pdf]

Table S1: Blast results for amplicons of the clinical samples and plasmid controls

No.	Sample Code	FECT	PCR	Sequencing primers	Sequence	Max. Score	E. value	Identity	Accession number of the maximum scored sequence
1	AsT7	plasmid control		T7-F, T7-R	GGTGATGTAATAGCAGTCGGCGGTTTCTTTTTTTT<br>TGGCGGACAATTGCATGCGATTTGCTATGTGTTG<br>AGGGAGAATAGGTGGCATGTTGGGCTTGTTAGAA<br>AGGCATGCCGCTAGCGCTTATTTTCCCGCTATTT<br>CGTAACAACGGTGTCCATTTTGGCGTCTACGCTT<br>CACCGAACTATCGCCTGGACCGTCGGTAGCGAT<br>[illegible]	399	2.00E-107	218/219(99%)	MK849920.1
						399	2.00E-107	218/219(99%)	LC422643.1
						399	2.00E-107	218/219(99%)	MF358963.1
						399	2.00E-107	218/219(99%)	HQ721819.1
						383	2.00E-102	215/219(98%)	AJ000895.1
2	D27	As	As	As-F, As-R	GGTGATGTAATAGCAGTCGGCGGTTTCTTTTTTTT<br>TGGCGGACAATTGCATGCGATTTGCTATGTGTTG<br>AGGGAGAATAGGTGGCATGTTGGGCTTGTTAGAA<br>AGGCATGGTAGCGCTTATTTTCCCGCTATTTCGTA<br>ACAACGGTGTCCATTTTGGCGTCTACGCTTCACC<br>GAGCTATCGCCTGGACCGTCGGTAGCGATGAAA<br>[illegible]	390	1.00E-104	215/217(99%)	AJ000895.1
						385	5.00E-103	216/219(99%)	MK849920.1
						385	5.00E-103	216/219(99%)	LC422643.1
						385	5.00E-103	216/219(99%)	MF358963.1
						385	5.00E-103	216/219(99%)	HQ721819.1
3	D21	As	As	As-F, As-R	GGTGATGTAATAGCAGTCGGCGGTTTCTTTTTTTT<br>TGGCGGACAATTGCATGCGATTTGCTATGTGTTG<br>CGGGAGAATAGGTGGCATGTTGGGCTTGTTAGAA<br>AGGCATGCGTAGCGCTTATTTTCCCGCTATTTCG<br>TAACAACGGTGTCCATTTTGGCGTCTACGCTTCA<br>CCGAGCTATCGCCTGGACCGTCGGTAGCGATGA<br>[illegible]	392	3.00E-105	215/217(99%)	AJ000895.1
						387	1.00E-103	216/219(99%)	MK849920.1
						387	1.00E-103	216/219(99%)	LC422643.1
						387	1.00E-103	216/219(99%)	MF358963.1
						387	1.00E-103	216/219(99%)	HQ721819.1
4	D25	As	As	As-F, As-R	GGTGATGTAATAGCAGTCGGCGGTTTCTTTTTTTT<br>TGGCGGACAATTGCATGCGATTTGCTATGTGTTG<br>AGGGAGAATAGGTGGCATGTTGGGCTTGTTAGAA<br>AGGCATGCGTAGCGCTTATTTTCCCGCTATTTCG<br>TAACAACGGTGTCCATTTTGGCGTCTACGCTTCA<br>CCGAACTATCGCCTGGACCGTCGGTAGCGATGAA<br>[illegible]	392	3.00E-105	215/217(99%)	AJ000895.1
						387	1.00E-103	216/219(99%)	MK849920.1
						387	1.00E-103	216/219(99%)	LC422643.1
						387	1.00E-103	216/219(99%)	MF358963.1
						387	1.00E-103	216/219(99%)	HQ721819.1
5	D53	NF	As+Ss+Hw	As-F, As-R	GGTGATGTAATAGCAGTCGGCGGTTTCTTTTTTTT<br>TGGCGGACAATTGCATGCGATTTGCTATGTGTTG<br>AGGGAGAATAGGAGTCATGTTGGGCTTGTTAGAA<br>AGGCATGCGTAGCGCTTATTTTCCCGCTATTTCG<br>TAACAACGGTGTCCATTTTGGCGTCTACGCTTCA<br>CCGAGCTATCGCCTGGACCGTCGGTAGCGATGA<br>[illegible]	387	1.00E-103	214/217(99%)	AJ000895.1
						381	6.00E-102	215/219(98%)	MK849920.1
						381	6.00E-102	215/219(98%)	LC422643.1
						381	6.00E-102	215/219(98%)	MF358963.1
						381	6.00E-102	215/219(98%)	HQ721819.1
6	D56	NF	As+Ss	As-F, As-R	GGTGATGTAATAGCAGTCGGCGGTTTCTTTTTTTT<br>TGGCGGACAATTGCATGCGATTTGCTATGTGTTG<br>AGGGAGAATAGGTGGCATGTTGGGCTTGTTAGAA<br>AGGCATGCGTAGCGCTTATTTTCCCGCTATTTCG<br>TAACAACGGTGTCCATTTTGGCGTCTACGCTTCA<br>CCGACTATCGCCTGGACCGTCGGTAGCGATGAAA<br>[illegible]	390	1.00E-104	215/217(99%)	AJ000895.1
						385	5.00E-103	216/219(99%)	MK849920.1
						385	5.00E-103	216/219(99%)	LC422643.1
						385	5.00E-103	216/219(99%)	MF358963.1
						385	5.00E-103	216/219(99%)	HQ721819.1
7	BK75	Hw	As+Hw	As-F, As-R	GGTGATGTAATAGCAGTCGGCGGTTTCTTTTTTTT<br>TGGCGGACAATTGCATGCGATTTGCTATGTGTTG<br>AGGGAGAATAGGTGTCATGTTGGGCTTGTTAGAA<br>AGGCATGCATAGCGCTTATTTTCCCGCTATTTCGT<br>AACAACGGTGTCCATTTTGGCGTCTACGCTTCAC<br>CGAGCTATCGCCTGGACCGTCGGTAGCGATGAA<br>[illegible]	390	1.00E-104	215/217(99%)	AJ000895.1
						381	6.00E-102	215/219(98%)	MK849920.1
						381	6.00E-102	215/219(98%)	LC422643.1
						381	6.00E-102	215/219(98%)	MF358963.1
						381	6.00E-102	215/219(98%)	HQ721819.1
8	Hw-T7	plasmid control		T7-F, T7-R	AGCATTGCTTGAATGCCGCCTCAATTTTTGTATTG<br>GTGGTTAGACACACACACATAACTTGTGTGGTGT<br>GGTACCTGTTCTTGTGATCAGGAAACCTTAATGAT<br>CCTTCACATGTTAACCAATAATGCGCGCTACGTG<br>TTATGGTGGATGGGACAATATGTGTGGACGCCAA<br>CACAAAATATTAACTTTTTACATTTGATGTTTGCAG<br>ATGATCGTGACTTCATCTTAGTCGTCACATTAGAC<br>TCGACTAGCTTCAGCGATGGATCGGTCGATTCGC	881	0.0	477/477 (100%)	KM891738.1
						881	0.0	477/477 (100%)	LC036565.1
						881	0.0	477/477 (100%)	LC036563.1
						876	0.0	476/477 (99%)	LC088287.1
						876	0.0	476/477 (99%)	AB793527.1
						870	0.0	475/477 (99%)	AF217891.1
9	22	NF	Hw+Ss	Hw-F,Hw-R	AGCATTGCTTGAATGCCGCCTCAATTTTTGTATTG<br>GTGGTTGGACACACACACATAACTTGTGTGGTGT<br>GGTACCTGTTCTTGTGATCAGGAAACCTTAATGAT<br>CCTTCACATGTTAACCAATAATGCGCGCTACGTG<br>TTATGGTGGATGGGACAATATGTGTGGACGCCAA<br>CACAAAATATTAACTTTTTACATTTGATGTTTGCAG<br>ATGATCGTGACTTCATCTTAGTCGTCACATTAGAC<br>TCGACTAGCTTCAGCGATGGATCGGTCGATTCGC	881	0.0	477/477 (100%)	LC088287.1
						881	0.0	477/477 (100%)	AB793527.1
						876	0.0	476/477 (99%)	KM891738.1
						876	0.0	476/477 (99%)	LC036565.1
						876	0.0	476/477 (99%)	LC036563.1
						876	0.0	476/477 (99%)	AF217891.1
10	D53	NF	As+Hw+Ss	Hw-F,Hw-R	AGCATTGCTTGAATGCCGCCTCAATTTTTGTATTG<br>GTGGTTGGACACACACACATAACTTGTGTGGTGT<br>GGTACCTGTTCTTGTGATCAGGAAACCTTAATGAT<br>CCTTCACATGTTAACCAATAATGCGCGCTACGTG<br>TTATGGTGGATGGGACAATATGTGTGGACGCCAA<br>CACAAAATATTAACTTTTTACATTTGATGTTTGCAG<br>ATGATCGTGACTTCATCTTAGTCGTCACATTAGAC<br>TCGACTAGCTTCAGCGATGGATCGGTCGATTCGC	881	0.0	477/477 (100%)	LC088287.1
						881	0.0	477/477 (100%)	AB793527.1
						876	0.0	476/477 (99%)	KM891738.1
						876	0.0	476/477 (99%)	LC036565.1
						876	0.0	476/477 (99%)	LC036563.1
						876	0.0	476/477 (99%)	AF217891.1
11	D64	NF	As+Hw+Ss	Hw-F,Hw-R	AGCATTGCTTGAATGCCGCCTCAATTTTTGTATTG<br>GTGGTTAGACACACACACATAACTTGTGTGGTGT<br>GGTACCTGTTCTTGTGATCAGGAAACCTTAATGAT<br>CCTTCACATGTTAACCAATAATGCGCGCTACGTG<br>TTATGGTGGATGGGACAATATGTGTGGACGCCAA<br>CACAAAATATTAACTTTTTACATTTGATGTTTGCAG<br>ATGATCGTGACTTCATCTTAGTCGTCACATTAGAC<br>TCGACTAGCTTCAGCGATGGATCGGTCGATTCGC	881	0.0	477/477 (100%)	KM891738.1
						881	0.0	477/477 (100%)	LC036565.1
						881	0.0	477/477 (100%)	LC036563.1
						876	0.0	476/477 (99%)	LC088287.1
						876	0.0	476/477 (99%)	AB793527.1
						870	0.0	475/477 (99%)	AF217891.1
12	D90	As+Hw	As+Hw	Hw-F,Hw-R	AGCATTGCTTGAATGCCGCCTCAATTTTTGTATTG<br>GTGGTTGGACACACACACATAACTTGTGTGGTGT<br>GGTACCTGTTCTTGTGATCAGGAAACCTTAATGAT<br>CCTTCACATGTTAACCAATAATGCGCGCTACGTG<br>TTATGGTGGATGGGACAATATGTGTGGACGCCAA	881	0.0	477/477 (100%)	LC088287.1
						881	0.0	477/477 (100%)	AB793527.1
						876	0.0	476/477 (99%)	KM891738.1
						876	0.0	476/477 (99%)	LC036565.1

					CACAAAATATTAACTTTTTACATTTGATGTTTGCAG<br>ATGATCGTGACTTCATCTTAGTCGTCACATTAGAC<br>TCGACTAGCTTCAGCGATGGATCGGTCGATTCGC	876	0.0	476/477 (99%)	LC036563.1
						876	0.0	476/477 (99%)	AF217891.1
13	D97	As+Hw	As+Hw	Hw-F,Hw-R	AGCATTGCTTGAATGCCGCCTCAATTTTTGTATTG<br>GTGGTTGGACACACACACATAACTTGTGTGGTGT<br>GGTACCTGTTCTTGTGATCAGGAAACCTTAATGAT<br>CCTTCACATGTTAACCAATAATGCGCGCTACGTG<br>TTATGGTGGATGGGACAATATGTGTGGACGCCAA<br>CACAAAATATTAACTTTTTACATTTGATGTTTGCAG<br>ATGATCGTGACTTCATCTTAGTCGTCACATTAGAC<br>TCGACTAGCTTCAGCGATGGATCGGTCGATTCGC	881	0.0	477/477 (100%)	LC088287.1
						881	0.0	477/477 (100%)	AB793527.1
						876	0.0	476/477 (99%)	KM891738.1
						876	0.0	476/477 (99%)	LC036565.1
						876	0.0	476/477 (99%)	LC036563.1
						876	0.0	476/477 (99%)	AF217891.1
14	Ss4-T7	plasmid control		T7-F, T7-R	GAATTCCAAGTAAACGTAAGTCATTAGCTTACATT<br>GATTACGTCCCTGCCCTTTGTACACACCGCCCGT<br>CGGCTGCCCGGAACTGAGCAATATCCAGACGCA	176	1.E-40	100/102 (98%)	M84229.1
						172	2.E-39	99/102 (97%)	AF036605.1
						171	6.E-39	99/102 (97%)	XM_024646606.1
						171	6.E-39	99/102 (97%)	LC038066.1
						171	6.E-39	99/102 (97%)	LM524975.1
						171	6.E-39	99/102 (97%)	LM524969.1
						171	6.E-39	99/102 (97%)	AB923889.1
						171	6.E-39	99/102 (97%)	AB923888.1
15	6	NF	Ss	Ss-F, Ss-R	GAATTCCAAGTAAACGTAAGTCATTAGCTTACATT<br>GATTACGTCCCTGCCCTTTGTACACACCGCCCGT<br>CGCTGCCCGGAACTGAGCAATATCCAGAGGCA	187	5.E-44	101/101 (100%)	M84229.1
						182	3.E-42	100/101 (99%)	XM_024646606.1
						182	3.E-42	100/101 (99%)	LC038066.1
						182	3.E-42	100/101 (99%)	LM524975.1
						182	3.E-42	100/101 (99%)	LM524969.1
						182	3.E-42	100/101 (99%)	AB923889.1
						182	3.E-42	100/101 (99%)	AB923888.1
16	3	NF	Ss	Ss-F, Ss-R	GAATTCCAAGTAAACGTAAGTCATTAGCTTACATT<br>GATTACGTCCCTGCCCTTTGTACACACCGCCCGT<br>CGCTGCCCGGAACTGAGCAATATCCAGAGGCA	187	5.E-44	101/101 (100%)	M84229.1
						182	3.E-42	100/101 (99%)	XM_024646606.1
						182	3.E-42	100/101 (99%)	LC038066.1
						182	3.E-42	100/101 (99%)	LM524975.1
						182	3.E-42	100/101 (99%)	LM524969.1
						182	3.E-42	100/101 (99%)	AB923889.1
						182	3.E-42	100/101 (99%)	AB923888.1
17	15	NF	Ss	Ss-F, Ss-R	GAATTCCAAGTAAACGTAAGTCATTAGCTTACATT<br>GATTACGTCCCTGCCCTTTGTACACACCGCCCGT<br>CGCTGCCCGGAACTGAGCAATATCCAGAGGCA	187	5.E-44	101/101 (100%)	M84229.1
						182	3.E-42	100/101 (99%)	XM_024646606.1
						182	3.E-42	100/101 (99%)	LC038066.1
						182	3.E-42	100/101 (99%)	LM524975.1
						182	3.E-42	100/101 (99%)	LM524969.1
						182	3.E-42	100/101 (99%)	AB923889.1
						182	3.E-42	100/101 (99%)	AB923888.1
18	8	NF	Ss	Ss-F, Ss-R	GAATTCCAAGTAAACGTAAGTCATTAGCTTACATT<br>GATTACGTCCTGCCCTTTGTACACACCGCCCGTC<br>GCTGCCCGGAACTGAGCAATATCCAGAGGCA	180	9.E-42	100/101 (99%)	M84229.1
						174	4.E-40	99/101 (98%)	XM_024646606.1
						174	4.E-40	99/101 (98%)	LC038066.1
						174	4.E-40	99/101 (98%)	LM524975.1
						174	4.E-40	99/101 (98%)	LM524969.1
						174	4.E-40	99/101 (98%)	AB923889.1
						174	4.E-40	99/101 (98%)	AB923888.1
19	27	Ss	Ss	Ss-F, Ss-R	GAATTCCAAGTAAACGTAAGTCATTAGCTTACATT<br>GATTACGTCCCTGCCCTTTGTACACACCGCCCGT<br>CGCTGCCCGGAACTGAGCAATATCCAGAGGCA	187	5.E-44	101/101 (100%)	M84229.1
						182	3.E-42	100/101 (99%)	XM_024646606.1
						182	3.E-42	100/101 (99%)	LC038066.1
						182	3.E-42	100/101 (99%)	LM524975.1
						182	3.E-42	100/101 (99%)	LM524969.1
						182	3.E-42	100/101 (99%)	AB923889.1
						182	3.E-42	100/101 (99%)	AB923888.1
20	33	Ss	Ss	Ss-F, Ss-R	GAATTCCAAGTAAACGTAAGTCATTAGCTTACATT<br>GATTACGTCCCTGCCCTTTGTAACACCGCCCGTC<br>GCTGCCCGGAACTGAGCAATATCCAGAGGCA	180	9.E-42	100/101 (99%)	M84229.1
						174	4.E-40	99/101 (98%)	XM_024646606.1
						174	4.E-40	99/101 (98%)	LC038066.1
						174	4.E-40	99/101 (98%)	LM524975.1
						174	4.E-40	99/101 (98%)	LM524969.1
						174	4.E-40	99/101 (98%)	AB923889.1
						174	4.E-40	99/101 (98%)	AB923888.1
21	28	Ss	Ss	Ss-F, Ss-R	GAATTCCAAGTAAACGTAAGTCATTAGCTTACATT<br>GATTACGTCCCTGCCcTTTGTACACACCGCCCGT<br>CGCTGCCCGGAACTGAGCAATATCCAGAGCA	182	2.E-42	98/98 (100%)	M84229.1
						176	1.E-40	97/98 (99%)	XM_024646606.1
						176	1.E-40	97/98 (99%)	LC038066.1
						176	1.E-40	97/98 (99%)	LM524975.1
						176	1.E-40	97/98 (99%)	LM524969.1
						176	1.E-40	97/98 (99%)	AB923889.1
						176	1.E-40	97/98 (99%)	AB923888.1
22	44	Hw	Ss	Ss-F, Ss-R	GAATTCCAAGTAAACGTAAGTCATTAGCTTACATT<br>GATTACGTCCCTGCCCTTTGTACACACCGCCCGT<br>CGCTGCCCGGAACTGAGCAATATCCAGAGGCA	187	5.E-44	101/101 (100%)	M84229.1
						182	3.E-42	100/101 (99%)	XM_024646606.1
						182	3.E-42	100/101 (99%)	LC038066.1
						182	3.E-42	100/101 (99%)	LM524975.1
						182	3.E-42	100/101 (99%)	LM524969.1
						182	3.E-42	100/101 (99%)	AB923889.1
						182	3.E-42	100/101 (99%)	AB923888.1

23	H19	Hw	Ss	Ss-F, Ss-R	GAATTCCAAGTAAACGTAAGTCATTAGCTTACATTGATTACGTCCCTGCCCTTTGTACACACCGCCCGTCGCTGCCCGGAACTGAGCAATATCCAGAGGCA	187	5.E-44	101/101 (100%)	M84229.1
						182	3.E-42	100/101 (99%)	XM_024646606.1
						182	3.E-42	100/101 (99%)	LC038066.1
						182	3.E-42	100/101 (99%)	LM524975.1
						182	3.E-42	100/101 (99%)	LM524969.1
						182	3.E-42	100/101 (99%)	AB923889.1
						182	3.E-42	100/101 (99%)	AB923888.1
24	D63	NF	As+Ss	Ss-F, Ss-R	GAATTCCAAGTAAACGTAAGTCATTAGCTTACATTGATTACGTCCCTGCCCTTTGTACACACCGCCCGTCGCTGCCCGGAACTGAGCAATATCCAGAGGCA	187	5.E-44	101/101 (100%)	M84229.1
						182	3.E-42	100/101 (99%)	XM_024646606.1
						182	3.E-42	100/101 (99%)	LC038066.1
						182	3.E-42	100/101 (99%)	LM524975.1
						182	3.E-42	100/101 (99%)	LM524969.1
						182	3.E-42	100/101 (99%)	AB923889.1
						182	3.E-42	100/101 (99%)	AB923888.1
25	D73	NF	As+Ss	Ss-F, Ss-R	GAATTCCAAGTAAACGTAAGTCATTAGCTTACATTGATTACGTCCCTGCCCTTTGTACACACCGCCCGTCGCTGCCCGGAACTGAGCAATATCCAGAGGCA	187	5.E-44	101/101 (100%)	M84229.1
						182	3.E-42	100/101 (99%)	XM_024646606.1
						182	3.E-42	100/101 (99%)	LC038066.1
						182	3.E-42	100/101 (99%)	LM524975.1
						182	3.E-42	100/101 (99%)	LM524969.1
						182	3.E-42	100/101 (99%)	AB923889.1
						182	3.E-42	100/101 (99%)	AB923888.1
26	D98	As	As+Ss	Ss-F, Ss-R	GAATTCCAAGTAAACGTAAGTCATTAGCTTACATTGATTACGTCCCTGCCCTTTGTACACACCGCCCGTCGCTGCCCGGAACTGAGCAATATCCAGAGGCA	187	5.E-44	101/101 (100%)	M84229.1
						182	3.E-42	100/101 (99%)	XM_024646606.1
						182	3.E-42	100/101 (99%)	LC038066.1
						182	3.E-42	100/101 (99%)	LM524975.1
						182	3.E-42	100/101 (99%)	LM524969.1
						182	3.E-42	100/101 (99%)	AB923889.1
						182	3.E-42	100/101 (99%)	AB923888.1
27	22	NF	Hw+Ss	Ss-F, Ss-R	GAATTCCAAGTAAACGTAAGTCATTAGCTTACATTGATTACGTCCCTGCCCTTTGTACACACCGCCCGTCGCTGCCCGGAACTGAGCAATATCCAGAGGCA	187	5.E-44	101/101 (100%)	M84229.1
						182	3.E-42	100/101 (99%)	XM_024646606.1
						182	3.E-42	100/101 (99%)	LC038066.1
						182	3.E-42	100/101 (99%)	LM524975.1
						182	3.E-42	100/101 (99%)	LM524969.1
						182	3.E-42	100/101 (99%)	AB923889.1
						182	3.E-42	100/101 (99%)	AB923888.1
28	D53	NF	As+Hw+Ss	Ss-F, Ss-R	GAATTCCAAGTAAACGTAAGTCATTAGCTTACATTGATTACGCACTGCCCTTTGTACACACCGCCCGTCGCTGCCCGGAACTGAGCAATATCCAGAGGCA	174	4.E-40	99/101 (98%)	M84229.1
						169	2.E-38	98/101 (97%)	XM_024646606.1
						169	2.E-38	98/101 (97%)	LC038066.1
						169	2.E-38	98/101 (97%)	LM524975.1
						169	2.E-38	98/101 (97%)	LM524969.1
						169	2.E-38	98/101 (97%)	AB923889.1
						169	2.E-38	98/101 (97%)	AB923888.1

FECT: Formalin ethyl acetate concentration technique
As: Ascaris lumbricoides
Hw: Hookworm
Ss: Strongyloides stercoralis
NF: Not found
